# Cyclopeptide RA-V Inhibits Organ Enlargement and Tumorigenesis Induced by YAP Activation

**DOI:** 10.3390/cancers10110449

**Published:** 2018-11-16

**Authors:** Xinyan Ji, Lihua Song, Li Sheng, Anhui Gao, Yang Zhao, Shixun Han, Yuchao Zhang, Chu Zhu, Simeng Zhao, Zhe Wang, Bohan Xu, Li Li, Jia Li, Ninghua Tan, Bin Zhao

**Affiliations:** 1 MOE Key Laboratory of Biosystems Homeostasis and Protection and Innovation Center for Cell Signaling Network, Life Sciences Institute, Zhejiang University, Hangzhou 310058, China; jixinyan@zju.edu.cn (X.J.); zhaoyang15@zju.edu.cn (Y.Z.); shixunhan@foxmail.com (S.H.); zhangyuchao0713@163.com (Y.Z.); zucu@zju.edu.cn (C.Z.); bohanxu@zju.edu.cn (B.X.); 2 School of Traditional Chinese Pharmacy and State Key Laboratory of Natural Medicines, China Pharmaceutical University, Nanjing 211198, China; songlihua4835@163.com (L.S.); wangzhe@cpu.edu.cn (Z.W.); 3 The National Center for Drug Screening, 189 Guoshoujing Road, Shanghai 201203, China; lsheng@simm.ac.cn (L.S.); ahgao@simm.ac.cn (A.G.); 4 State Key Laboratory of Phytochemistry and Plant Resources in West China, Kunming Institute of Botany, Chinese Academy of Sciences, Kunming 650201, China; liuzhengyirs@126.com; 5 Institute of Aging Research, Hangzhou Normal University, Hangzhou 311121, China; lili@hznu.edu.cn; 6 State Key Laboratory of Drug Research, Shanghai Institute of Materia Medica, Chinese Academy of Sciences, Shanghai 201203, China

**Keywords:** hippo pathway, YAP, TAZ, RA-V, cancer, organ size, protein synthesis

## Abstract

The Hippo pathway restricts organ size during development and its inactivation plays a crucial role in cancer. Yes-associated protein (YAP) and its paralog transcriptional coactivator with PSD-95/Dlg/ZO-1 (PDZ)-binding motif (TAZ) are transcription co-activators and effectors of the Hippo pathway mediating aberrant enlargement of organs and tumor growth upon Hippo pathway inactivation. It has been demonstrated that genetic inactivation of YAP could be an effective approach to inhibit tumorigenesis. In order to identify pharmacological inhibitors of YAP, we screened a library of 52,683 compounds using a YAP-specific reporter assay. In this screen we identified cyclopeptide RA-V (deoxybouvardin) as a specific inhibitor of YAP and TAZ but not other reporters. Unexpectedly, later experiments demonstrated that RA-V represses the protein but not mRNA levels of YAP target genes. Nevertheless, RA-V strongly blocks liver enlargement induced by *Mst1/2* knockout. Furthermore, RA-V not only inhibits liver tumorigenesis induced by YAP activation, but also induces regression of established tumors. We found that RA-V inhibits dedifferentiation and proliferation, while inducing apoptosis of hepatocytes. Furthermore, RA-V also induces apoptosis and inhibits proliferation of macrophages in the microenvironment, which are essential for YAP-induced tumorigenesis. RA-V is thus a drug candidate for cancers involving YAP/TAZ activation.

## 1. Introduction

The Hippo pathway is a key mechanism for organ size control identified during *Drosophila* genetic screens [1,2]. Through transcriptional regulation of gene expression, the Hippo pathway controls cell proliferation, apoptosis, and stem cell self-renewal, thus determining cell number in certain tissues or organs. Its inactivation leads to drastic enlargement of organs in both *Drosophila* and mammals. The Hippo pathway is formed by the Sterile 20 (STE-20) family kinases serine/threonine kinase 4/3 (STK4/3, also called MST1/2) and the downstream protein kinase A, G, and C (AGC) family kinases large tumor suppressor kinase 1/2 (LATS1/2). MST1/2 activates LATS1/2 by directly phosphorylating LATS1/2 and by phosphorylating adaptor proteins salvador family WW domain containing protein 1 (SAV1) and MOB kinase activator 1A/B (MOB1A/B). LATS1/2 then phosphorylates transcription co-activators Yes-associated protein (YAP) and transcriptional coactivator with PDZ-binding motif (TAZ) on multiple HXRXXS motifs [3,4,5,6,7]. Phosphorylation of YAP S127 promotes binding to scaffold protein 14-3-3, which leads to cytoplasmic retention of YAP [5]. Furthermore, phosphorylation of YAP S381 primes further phosphorylation by casein kinase 1, and then recruitment of E3 ligase SCF^β-TRCP^ for ubiquitination and degradation [8]. When the Hippo pathway is inactive, YAP/TAZ translocate to the cell nucleus and interact with transcription factors such as the TEA domain (TEAD) family proteins to induce gene expression [9,10,11,12,13].

The potent activity of YAP in promoting cell proliferation and inhibiting apoptosis suggests a role of the Hippo pathway in cancer. Indeed, massive tumorigenesis was observed in liver-specific *Mst1/2* knockout or *Yap* transgenic mice [3,14,15,16,17,18]. Interestingly, *Yap* activation also promotes liver tumorigenesis through inducing hepatocyte dedifferentiation [19] and non-cell-autonomously recruiting type 2 macrophages to protect tumor-initiating cells [20,21]. In human cancers, mutations of Hippo pathway upstream components lead to YAP activation. For instance, mutation of *neurofibromin 2* (*NF2*) is a major reason for the development of neurofibromatosis type 2. Remarkably, inactivation of only one allele of *Yap* blocks liver tumorigenesis induced by *Nf2* knockout in mice [22]. Furthermore, activating mutations of *GNAQ* or *GNA11*, which encode G protein alpha subunits G_αq_ and G_α11_, have been identified in about 80% of uveal melanomas. Recent studies showed that by mediating G protein-coupled receptor (GPCR) signaling, G_αq_ and G_α11_ strongly activate YAP, and inhibition of YAP could repress tumorigenesis of uveal melanoma [23,24]. Moreover, the YAP gene locus is amplified in a few human cancers such as hepatocellular carcinoma (HCC) and squamous cell carcinoma (SCC) [25,26]. YAP activation promotes the proliferation of epidermal stem cells and epithelial–mesenchymal transition (EMT), thus regulating skin regeneration and tumorigenesis [27,28,29]. Several other mechanisms such as gene fusion, transcriptional deregulation, and mutation of other components have also been linked to YAP activation in human cancers [30]. Importantly, YAP not only promotes tumor initiation and growth, but also plays key roles in metastasis, drug resistance, and cancer relapse [30]. These findings raise the possibility of the Hippo pathway and YAP as targets for cancer therapy.

The potential of YAP inhibition as a cancer therapy is supported by genetic evidence. Since the function of YAP largely depends on TEAD family transcription factors, the main stream has been focusing on disrupting YAP–TEAD interaction. It was found that expression of a dominant-negative TEAD potently suppressed liver tumorigenesis resulting from *Yap* overexpression or *Nf2* inactivation [31]. Furthermore, by screening of more than 3300 FDA-approved or in-clinical-trial compounds, verteporfin (VP) was found to disrupt YAP–TEAD interaction and to suppress liver overgrowth [31]. However, verteporfin has general cellular toxicity and low aqueous solubility. In an independent direction, based on the crystal structures of TEAD–YAP and TEAD–VGLL4 (a co-factor competing with YAP for TEAD binding) complexes, a polypeptide was designed to occupy the binding pockets of YAP and VGLL4 on TEAD, thus blocking YAP binding [32]. This polypeptide, called “super-TDU”, has been shown to inhibit gastric cancer induced by *Helicobacter pylori* in mice. Furthermore, it was also reported that statins, a class of cholesterol-lowering drugs, suppress nuclear accumulation of YAP/TAZ by inhibiting the mevalonate–RHO pathway [33]. Of note, studies in animal models suggest a tumor suppressive function of statins in a wide variety of cancers [34].

Here we report the identification of cyclopeptide RA-V (deoxybouvardin) as an inhibitor of the protein levels of YAP target genes. RA-V induces apoptosis and inhibits proliferation of both hepatocytes and immune cells in the liver and inhibits dedifferentiation of hepatocytes. Therefore, RA-V strongly blocks liver enlargement and tumorigenesis induced by Hippo pathway ablation or YAP activation in vivo. Based on the results of our study, we suggest RA-V as a potential therapy for cancers involving YAP/TAZ activation.

## 2. Results

### 2.1. Identification of RA-V as An Inhibitor of YAP Reporter

**Figure 1:**
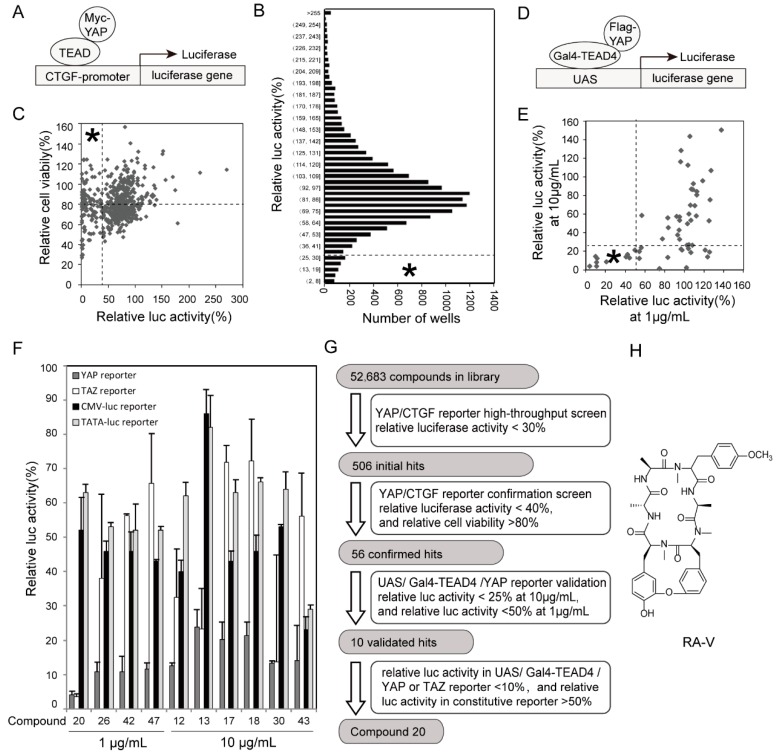
Identification of RA-V as an inhibitor of YAP (Yes-associated protein) reporter. (**A**) Illustration of the CTGF reporter system used in the primary screen. (**B**) Summary of the primary screen. Relative luciferase activities were calculated by normalization to dimethyl sulfoxide (DMSO)-treated control, and the distribution of activities was plotted. Asterisk represents the potion defined as positive. (**C**) Confirming screen using the same reporter assay with additional cell viability assay. Compounds were used at 10 μg/mL. Asterisk represents the potion defined as positive. (**D**) Illustration of the Gal4-TEAD4/9 × UAS-luciferase reporter assay used in the secondary screen. (**E**) Summary of the secondary screen. Luciferase activity was first normalized with β-galactosidase activity, and then relative activity was calculated by normalization to DMSO-treated control. Asterisk represents the potion defined as positive. (**F**) Validation of Compound 20 as a specific inhibitor of YAP and TAZ reporters. YAP and TAZ reporter assays are similar to that in (**D**). CMV and TATA driven reporters were used as controls. (**G**) Flowchart of the screen. (**H**) Chemical structure of RA-V.

In order to screen for YAP inhibitors, we established a HEK293T stable cell line expressing Myc-tagged YAP and a luciferase reporter gene driven by the promoter of *connective tissue growth factor* (*CTGF*), a well-established YAP target gene [9]. To reduce background, we used a minimal 250 bp *CTGF* promoter containing all TEAD binding sites. In this assay, the binding of YAP to the reporter gene is mediated by endogenous TEAD (Figure 1A). We chose a clone exhibiting more than 800,000-fold reporter activation (4D10) (Figure S1A). Short hairpin RNA (shRNA)-mediated knockdown of YAP or VP treatment inhibited the reporter activity (Figure S1B,C), which confirmed the establishment of a faithful YAP activity assay. Using this reporter cell, we screened a collection of 52,683 compounds including synthesized chemicals, natural products, and microbial secondary metabolites at a concentration of 10 μg/mL. In this screen, we identified 550 wells, representing 506 compounds (0.96% of the whole collection), which inhibited the reporter activity by more than 70% (Figure 1B). These 506 compounds went through a duplicate screen using the same reporter cell. At the same time, a cell viability assay was done to exclude non-specific cytotoxic compounds. From this duplicate screen we identified 56 compounds that consistently inhibited the reporter by more than 60%, while cell viability remained above 80% (Figure 1C). To screen for the most potent and specific hits from these candidates we used a second assay employing the 9 × UAS-luciferase reporter, which is driven by nine Gal4-binding elements. Cells were co-transfected with the reporter, TEAD4 fused with Gal4-DNA binding domain, and Flag-tagged YAP (Figure 1D). This assay has been previously reported as a specific assay for YAP activity [9]. Ten compounds inhibited the reporter activity by 75% at 10 μg/mL and by 50% at 1 μg/mL (Figure 1E). Since YAP and TAZ share similar transcriptional and regulatory mechanisms, we speculated that a specific YAP inhibitor would also inhibit TAZ but not other unrelated reporters. We therefore carried out a counter-screen using either YAP or TAZ in the Gal4-TEAD4/9 × UAS-luciferase reporter assay, and two unrelated reporters driven, respectively, by the cytomegalovirus (CMV) promoter and the TATA box. This experiment led to the identification of Compound 20 as the most potent and specific inhibitor of YAP and TAZ reporters (Figure 1F). The screen is summarized in Figure 1G. The identity of Compound 20 is RA-V (deoxybouvardin), a natural cyclopeptide first isolated from the roots of *Bouvardia ternifolia* and with a molecular formula of C_40_H_48_N_6_O_9_ [35] (Figure 1H). RA-V has been shown to inhibit tumor growth, angiogenesis, and inflammation, and to induce apoptosis [36,37,38], which would make sense if it inhibits YAP.

### 2.2. RA-V Represses the Protein Levels but Not mRNA Levels of YAP Target Genes

**Figure 2:**
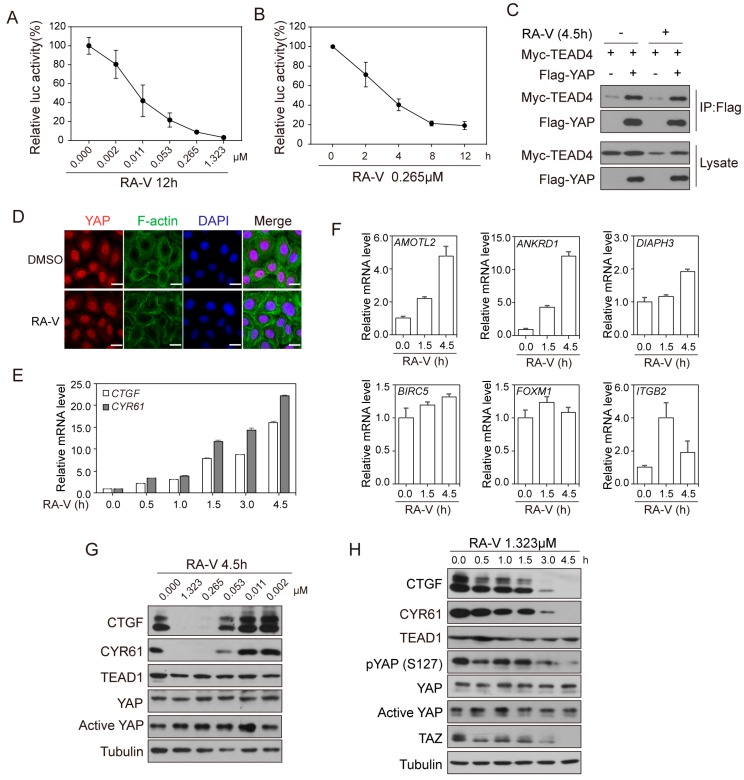
RA-V represses the protein levels but not mRNA levels of YAP target genes. (**A**,**B**) RA-V inhibits YAP reporter activity in a dose- (**A**) and time-dependent (**B**) manner. HEK293T cells were transfected with YAP, Gal4-TEAD4, 9 × UAS-luciferase, β-galactosidase and treated as the indicated. Luciferase activities were then measured and normalized to β-galactosidase activities. (**C**) RA-V does not affect YAP–TEAD interaction. HEK293A cells were transfected as indicated and treated with DMSO or RA-V at 0.265 μM for 4.5 h before harvest. Cell lysates were immunoprecipitated with anti-Flag antibody and examined by western blotting. The beads had a low-level non-specific binding of Myc-TEAD4. (**D**) RA-V does not affect YAP subcellular localization. HeLa cells were trypsinized and attached again in the presence of DMSO or RA-V at 0.265 μM for 4.5 h. Cells were then subjected to immunofluorescence staining. Scale bars, 20 μm. (**E**,**F**) RA-V induces mRNA expression of canonical YAP target genes *CTGF* and *CYR61* (**E**) as well as several other genes (**F**). HEK293A cells were trypsinized and attached again in the presence of DMSO or 1.323 μM RA-V for the indicated time. mRNA was extracted and gene expression levels were determined by quantitative RT-PCR. (**G**,**H**) The protein levels of CTGF and CYR61 were inhibited by RA-V in a dose- (**G**) and time-dependent (**H**) manner. HeLa and HEK293A cells were trypsinized and attached again for 4.5 h in the presence of DMSO or RA-V. Cells were then lysed and examined by western blotting. Values represent mean ± SD from three technical repeats.

In order to determine how RA-V inhibits the YAP reporter activity, we first confirmed that RA-V inhibited YAP reporter in a dose- and time-dependent manner (Figure 2A,B). When treated for 12 h, the IC50 of RA-V was about 10 nM. Repression of YAP activity could be due to the disruption of YAP–TEAD interaction or defective YAP nuclear localization. However, by co-immunoprecipitation assay, the interaction of YAP and TEAD was shown to be not affected by RA-V (Figure 2C). Furthermore, at a concentration of 0.265 μM (0.2 μg/mL) for 4.5 h, which resulted in 50% inhibition of the reporter activity, YAP nuclear localization was not affected in several cell lines (Figure 2D and Figure S2A). Consistently, when the phosphorylation-deficient YAP-5SA or TAZ-S89A mutants were used in the reporter assay, they were still inhibited by RA-V (Figure S2B), indicating a phosphorylation-independent mechanism. We therefore examined whether the mRNA levels of endogenous YAP target genes could be inhibited by RA-V. Surprisingly, in both HEK293A and HeLa cells, RA-V induced canonical YAP target genes *CTGF* and *CYR61* on the mRNA level in a time-dependent manner (Figure 2E and Figure S2C). We thus examined more YAP targets and found that several other genes such as *AMOTL2*, *ANKRD1*, *DIAPH3*, and *ITGB2* were also induced, while *BIRC5* and *FOXM1* were largely unchanged (Figure 2F). However, when the protein levels of YAP target genes were examined we found that both CTGF and CYR61 were inhibited by RA-V in a dose- and time-dependent manner in several cell lines (Figure 2G,H and Figure S2D,E). The protein levels of TEAD1, YAP and dephosphorylated active YAP remained constant although the protein level of TAZ was sometimes also repressed by RA-V (Figure 2G,H and Figure S2D,E). Bouvardin, an analog of RA-V with only one hydroxyl group difference, has been shown to inhibit protein synthesis at the level of 80S ribosome at a site involved in interaction with EF1 and EF2 [39]. Therefore, RA-V may selectively inhibit the synthesis of some proteins, for example, CTGF and CYR61, but not TEAD1 or YAP. However, this is unlikely the only mechanism to inhibit YAP target genes because RA-V inhibited luciferase activity in the YAP/TAZ reporter assay, but not in constitutive luciferase reporters (Figure 1F) or Wnt signaling reporter TOP-flash (Figure S2F), despite the fact that all these reporters are based on the firefly luciferase protein. Therefore, although the mechanism by which RA-V inhibits the protein levels of YAP target genes is not completely clear and could be complicated, it may involve the inhibition of protein synthesis.

### 2.3. RA-V Inhibits Liver Enlargement Induced by Hippo Pathway Inactivation

**Figure 3:**
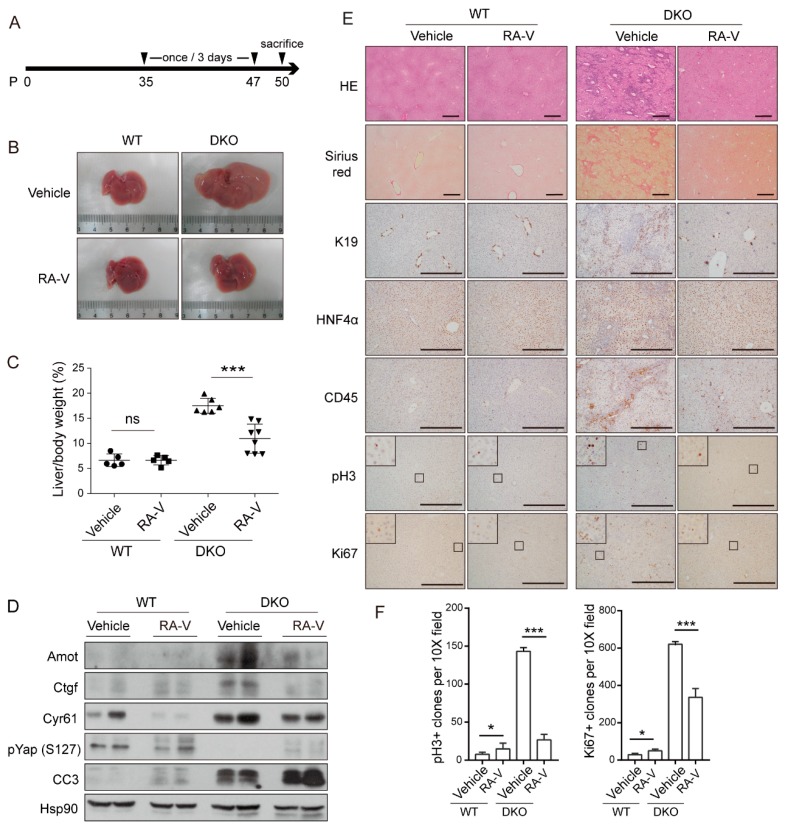
RA-V inhibits liver enlargement induced by Hippo pathway inactivation. (**A**) Schedule of vehicle or RA-V (10 mg/kg) injection. P: postnatal day. (**B**) RA-V inhibits liver enlargement in *Mst1/2* double knockout (DKO) mice. Images are representative of at least 5 mice. (**C**) Statistical analysis of the data in (B). Values represent mean ± SD with all data points plotted. (**D**) RA-V represses the protein levels of YAP target genes and induces apoptosis in liver. Liver tissue samples from two mice in each group were analyzed by western blotting. (**E**) Pathological and immunohistochemical (IHC) staining of liver sections from (B). Results are representative of at least five mice. Scale bars, 500 μm. Squared regions were enlarged for detailed view. (**F**) Quantification of pH3 (left panel) and Ki67 (right panel) positive cells. Six representative views were quantified. Experiments were duplicated. *p* values were determined by Student’s *t* test. ns, not significant; *, *p* < 0.1; ***, *p* < 0.001.

Since RA-V inhibits the expression of YAP target genes on the protein level, we examined whether it could inhibit the function of YAP in organ size control in vivo. It was reported that *Mst1/2* knockout strongly activates YAP and causes marked enlargement of the liver [14,18]. By *Albumin*-Cre-mediated liver-specific double knockout of *Mst1/2* (DKO) we confirmed a 3–4-fold enlargement of the liver at 2 months of age followed by tumorigenesis from 3 months (Figure S3A,B). RA-V has low solubility in aqueous solutions. In order to increase its bio-availability in vivo, RA-V was loaded in micelles as previously described [38]. RA-V micelles were intravenously injected at 10 mg/kg every 3 days from postnatal day 35 for a total of 5 times (Figure 3A). Mice were then sacrificed and analyzed. Injection of RA-V markedly inhibited liver enlargement in DKO mice (Figure 3B,C). Western blotting of liver lysates indicated that the protein levels of YAP target genes such as *Ctgf*, *Cyr61*, and *Amot* were induced by *Mst1/2* DKO and were inhibited by RA-V (Figure 3D). Furthermore, as previously reported, RA-V also promoted apoptosis as indicated by the enhanced cleaved caspase-3 (CC3) (Figure 3D). Interestingly, although RA-V does not enhance YAP phosphorylation in vitro, it promotes Yap phosphorylation in liver tissue (Figure 3D), likely through an indirect mechanism. Pathological analysis and immunohistochemical (IHC) staining of DKO liver sections demonstrated clear regions of hepatocyte dedifferentiation (gain of progenitor cell marker K19 and loss of hepatocyte marker hepatic nuclear factor 4 alpha, HNF4α) accompanied by leukocyte recruitment (indicated by CD45 staining) and fibrosis (indicated by Sirius red staining) (Figure 3E), which were consistent with previous reports. Remarkably, these phenotypes were largely reversed by RA-V treatment (Figure 3E). In addition, cell proliferation as indicated by phospho-histone H3 (pH3) and Ki67 staining was markedly inhibited by RA-V (Figure 3E,F). These data indicate that RA-V is an effective inhibitor of organ size enlargement induced by Hippo pathway inactivation, and it does this by inducing apoptosis and inhibition of both cell proliferation and dedifferentiation.

### 2.4. RA-V Inhibits Proliferation of Both Hepatocytes and Immune Cells

**Figure 4:**
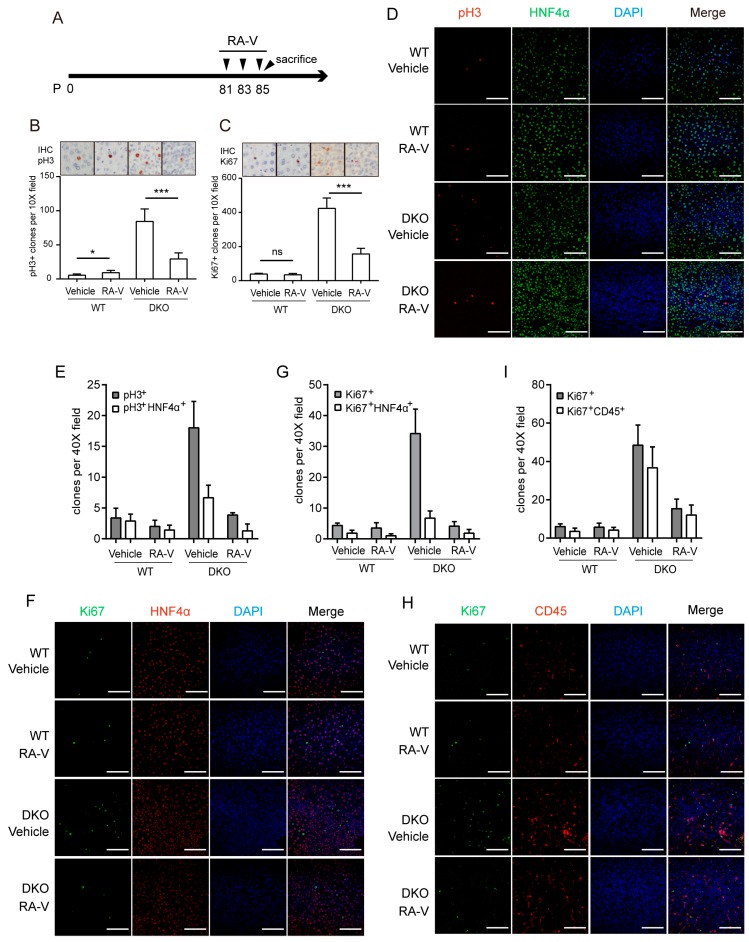
RA-V inhibits proliferation of both hepatocytes and immune cells. (**A**) Schedule of RA-V injection. (**B**,**C**) IHC staining and quantification of pH3 (**B**) and Ki67 (**C**) positive cells in liver sections from mice treated as (**A**). Images are representative of at least four mice. Six representative views were quantified. Values represent mean ± SD. *p* values were determined by Student’s *t* test. ns, not significant; ***, *p* < 0.001. (**D**–**I**) Multiplex immunohistochemical staining and quantification of pH3 and HNF4α (**D** and **E**), Ki67 and HNF4α (**F** and **G**), Ki67 and CD45 (**H** and **I**). Seven views in liver sections from two or three mice for each group were quantified. Values represent mean ± SD. Scale bars, 100 μm.

The Hippo pathway promotes liver enlargement and tumorigenesis through both cell-autonomous and non-cell-autonomous mechanisms. It was recently reported that recruitment of macrophages by YAP active cells promotes their survival and the ensuing tumorigenesis [20,21]. In order to determine whether RA-V directly inhibits hepatocyte proliferation or affects hepatocytes indirectly by modulating the immune microenvironment, we designed a short-term treatment and examined the responses of both hepatocytes and macrophages. RA-V was given at 5 mg/kg every other day only three times (Figure 4A). This short-term treatment did not significantly affect the liver/body weight ratio (Figure S4). However, it markedly inhibited cell proliferation induced by *Mst1/2* DKO as indicated by both pH3 and Ki67 staining (Figure 4B,C). We noticed that pH3 staining marked more cells in size and shape close to hepatocytes, and a large fraction of Ki67-positive cells are smaller cells resembling macrophages (Figure 4B,C). Indeed, multiplex immunohistochemistry indicated that about 37% of pH3-positive cells in *Mst1/2* DKO liver were HNF4α-positive (Figure 4D,E). It should not be forgotten that those dedifferentiated hepatocytes were not included. However, only 20% of Ki67-positive cells in *Mst1/2* DKO liver were also positive for HNF4α (Figure 4F,G). Consistently, 75% of Ki67-positive cells in *Mst1/2* DKO livers were CD45 positive (Figure 4H,I), confirming their leukocyte identity. The reason for the differential labeling of proliferating hepatocytes and immune cells by pH3 and Ki67 is unclear. Nevertheless, this staining indicated that both pH3^+^HNF4α^+^-proliferating hepatocytes and Ki67^+^CD45^+^-proliferating immune cells induced by *Mst1/2* DKO were largely reduced by short-term RA-V treatment (Figure 4D–I). These data suggest that RA-V suppresses liver enlargement of DKO mice by inhibiting proliferation of both hepatocytes and immune cells in the microenvironment.

### 2.5. RA-V Inhibits Liver Tumorigenesis in Mst1/2 Knockout Mice

**Figure 5:**
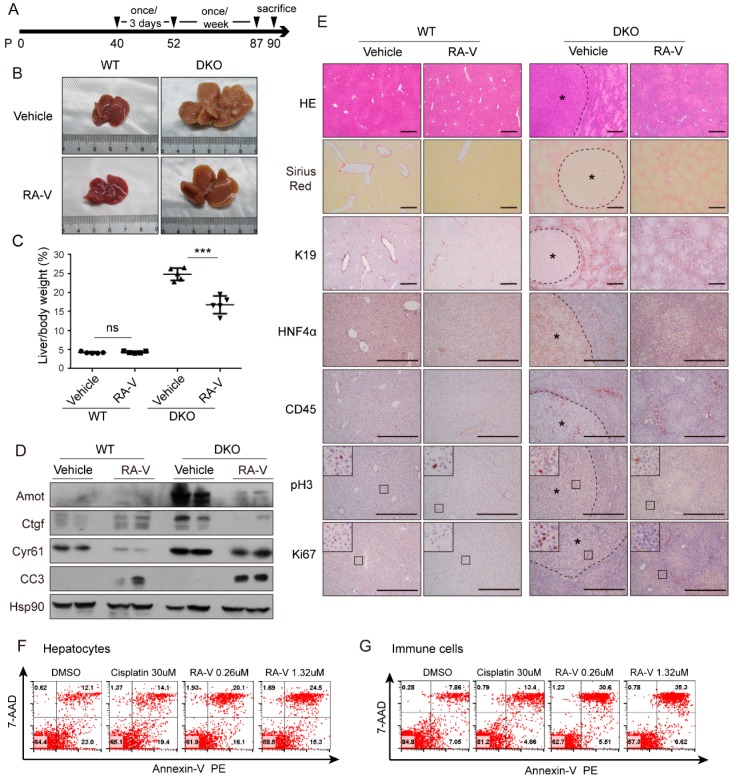
RA-V inhibits liver tumorigenesis in *Mst1/2* double knockout mice. (**A**) Schedule of RA-V (10 mg/kg) injection. (**B**,**C**) RA-V inhibits tumorigenesis in *Mst1/2* DKO mice. Images are representative of five mice (**B**). The live/body weight ratios were quantified (**C**) and values represent mean ± SD with all data points plotted. *p* values were determined by Student’s *t* test. ns, not significant; ***, *p* < 0.001. (**D**) RA-V represses protein levels of YAP target genes and promotes apoptosis in *Mst1/2* DKO livers. Two representative livers from each group were analyzed by western blotting. (**E**) Pathological and IHC staining of liver sections from (**B**). Results are representative of five mice. Scale bars, 500 μm. Asterisks denote tumors. (**F**,**G**) RA-V induces apoptosis of hepatocytes (**F**) and immune cells (**G**) isolated from tumors of *Mst1/2* DKO livers. Tumors were digested and fractionated by density gradient centrifugation. Respective cells were cultured in vitro for 12 h with DMSO, RA-V or cisplatin as indicated. Cells were then analyzed by flow cytometry. Experiments were duplicated.

A key application of a YAP inhibitor would be as an anticancer drug. Since RA-V exhibited potent activity in suppressing liver enlargement induced by Hippo pathway inactivation, we examined whether it could also inhibit tumorigenesis in the same context. RA-V was injected according to the same schedule as that in Figure 5A. Remarkably, while vehicle-treated *Mst1/2* DKO mice exhibited massive tumorigenesis, the RA-V-treated group was free of tumors when examined by the naked eye (Figure 5B). In this experiment, the liver/body weight ratio in the RA-V-treated group was much lower than that in the vehicle group but was still significantly higher than in wild-type mice (Figure 5C). This is either due to the lower frequency of drug injection in the last 40 days before sacrifice, or due to incomplete inhibition of YAP downstream gene expression. However, we confirmed that the protein levels of YAP target genes *Amot*, *Ctgf*, and *Cyr61* were effectively repressed by RA-V in this experiment (Figure 5D). By pathological analysis we found that tumors induced by *Mst1/2* DKO were highly proliferative and immune cell-infiltrated (Figure 5E). However, RA-V-treated livers were free of lesions, and dedifferentiation of hepatocytes in the para-tumor regions was partially alleviated (Figure 5E). We further found that in this experimental condition, RA-V clearly induced apoptosis in the liver tissue (Figure 5D). Moreover, RA-V treatment of both hepatocytes and immune cells isolated from the *Mst1/2* DKO tumors significantly induced apoptosis in a concentration much lower than that needed when using cisplatin, a drug commonly used for chemotherapy (Figure 5F,G). Therefore, RA-V is an effective inhibitor of tumorigenesis induced by Hippo pathway inactivation, likely through direct targeting of tumor-initiating cells and modulation of the immune microenvironment.

### 2.6. RA-V Induces Regression of Liver Tumors Induced by YAP Activation

**Figure 6:**
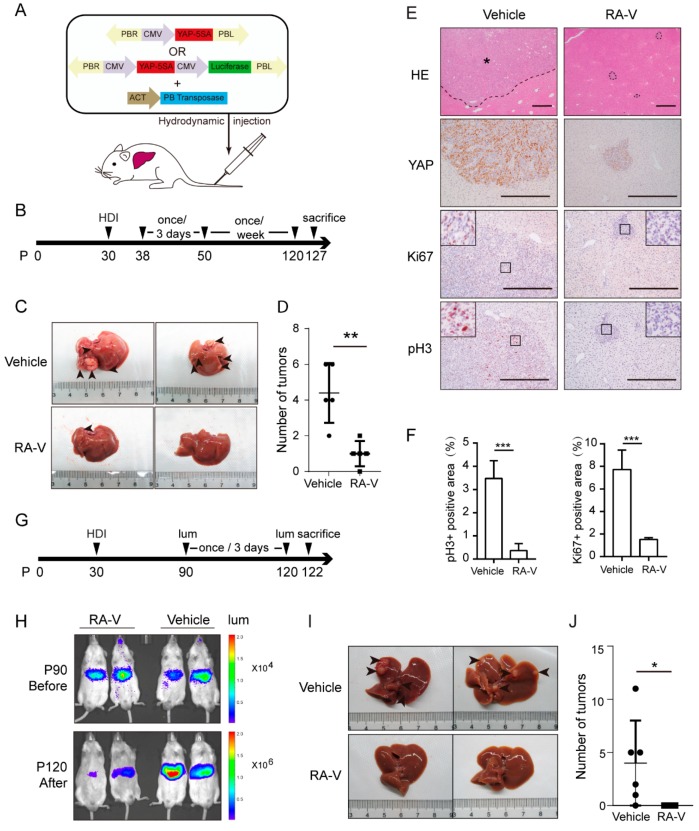
RA-V induces regression of liver tumors induced by YAP activation. (**A**) Scheme of liver tumorigenesis mouse model by hydrodynamic injection (HDI) of transposon plasmids. (**B**) Schedule of RA-V (5 mg/kg) injection. (**C**,**D**) RA-V inhibits liver tumorigenesis induced by expression of YAP-5SA. Images were representative livers from five mice (**C**). Arrowheads indicate tumors. Observable tumors were quantified (**D**). Values represent mean ± SD with all data points plotted. *p* values were determined by Student’s *t* test. **, *p* < 0.01. (**E**,**F**) RA-V markedly reduces lesion size and proliferation. Respective liver sections were stained (**E**), and cell proliferation was quantified by calculating the area of pH3/Ki67 positive signals relative to lesion area from eight fields by ImageJ (**F**). Scale bars, 500 μm. Asterisk denotes tumor. Values represent mean ± SD. *P* values were determined by Student’s *t* test. ***, *p* < 0.001. (**G**) Schedule of RA-V (10 mg/kg) treatment. (**H**–**J**) RA-V induces tumor regression. Mouse models were established and treated as in (**G**). Bioluminescence imaging was performed before and after RA-V treatment. Two representative mice from each group were shown (**H**). Livers were also dissected and pictured (**I**). Observable tumors were quantified (**J**). Values represent mean ± SD with all data points plotted. *P* values were determined by Student’s *t* test. *, *p* < 0.1.

YAP is the major effector of the Hippo pathway. We therefore examined whether RA-V could also inhibit tumorigenesis induced by expression of YAP. We have previously reported that expression of active YAP-5SA (serine-to-alanine mutation at all five Hippo pathway phosphorylation sites) mutant in mouse hepatocytes by hydrodynamic delivery induces liver tumors within three months [20] (Figure 6A). Based on this model, we designed an RA-V treatment schedule (Figure 6B). Similar to in the *Mst1/2* DKO mice, RA-V drastically repressed tumorigenesis (Figure 6C,D). Pathological analysis revealed much smaller lesions in livers treated with RA-V (Figure 6E). Furthermore, staining of pH3 and Ki67 indicated a much lower cell proliferation rate in lesions found in the RA-V-treated group (Figure 6F).

To further determine whether RA-V could induce regression of established tumors. We constructed a *Piggybac* transposon plasmid that co-expresses YAP-5SA and firefly luciferase. Tumors could thus be monitored by bioluminescent imaging. Hydrodynamic injection was used to perform as the above experiment, and tumors were detected by imaging after two months. RA-V was then injected every three days for a total of 10 times (Figure 6G). Subsequent imaging indicated partial regression of tumors in the RA-V-treated group and progression of tumors in the control group (Figure 6H). Regression of tumors was also confirmed by dissection of livers after sacrifice (Figure 6I,J). Therefore, RA-V is not only capable of inhibiting tumorigenesis but could also induce regression of YAP-induced tumors.

## 3. Discussion

YAP is a potent promoter of growth and inhibitor of apoptosis. Therefore, it is not surprising that deregulation of the Hippo pathway and activation of YAP strongly induces tumorigenesis [30]. Furthermore, activation of YAP/TAZ also promotes dedifferentiation and stemness; thus, it has also been linked to the maintenance of cancer stem cells [19,40]. Therefore, targeting of YAP/TAZ may be able to eradicate tumors from the root. The functions of the Hippo pathway in cancer have been most extensively examined in the context of liver cancer. It has been found that the *Yap* gene locus is amplified in mouse liver cancer [25]. A series of reports demonstrated that knockout of many Hippo pathway genes or transgenic expression of *Yap* potently induces liver tumorigenesis [1]. More intriguingly, YAP promotes liver tumorigenesis not only in a cell-autonomous manner, but also non-cell-autonomously by modulating the immune microenvironment [20,21]. YAP activates expression of cytokines and chemokines such as C-C motif chemokine ligand 2 (CCL2) and colony stimulating factor 1 (CSF1), thus recruiting macrophages to tumor-initiating cells to protect them from immunosurveillance. Therefore, YAP-induced tumorigenesis might be repressed by directly targeting tumor cells or by normalization of the immune microenvironment. Disruption of the interaction between YAP and its transcription factor partner TEADs has been a major direction for the development of YAP inhibitors [31,32]. However, pharmacological targeting of protein–protein interactions has proven challenging. Although previous studies have identified inhibitors such as VP and super-TDU, they are yet to be proven safe and potent enough for therapeutic use. Interestingly, statins, a class of cholesterol-lowering drugs, indirectly inhibit YAP/TAZ by affecting the membrane localization of Rho family GTPases [33]. In this report we found that RA-V potently inhibits YAP-induced organ size enlargement and liver tumorigenesis. Importantly, RA-V is able to induce regression of established tumors, which has not been shown for previous YAP inhibitors. We found that RA-V may function by simultaneously repressing tumor cells and macrophages in the immune microenvironment, thus crippling both mechanisms by which YAP promotes tumorigenesis.

RA-V is a natural product mainly purified from the roots and rhizomes of Rubia plants [41]. The roots of *Rubia cordifolia* (Chinese name: “Qian-Cao”) are widely used as a traditional Chinese medicine for the treatment of tuberculosis, rheumatism, contusion, and menoxenia [38]. RA-V has a unique bicyclic structure, and several mechanisms had been identified through which it may affect cellular activities. For example, it may suppress inflammation and tumor growth by targeting TAK1 and interrupting TAK1–TAB2 interaction in the NF-κB signaling pathway [38]. It could also inhibit ERK1/2 phosphorylation and the PI3K/AKT pathway thus affecting cell survival and migration [36,37]. Importantly, bouvardin, a very close derivative of RA-V, has been shown to inhibit protein synthesis. It binds to the 80S ribosome at a site involved in interactions with EF1 and EF2, thus inhibiting EF1-dependent binding of aminoacyl-tRNA and EF2-dependent translocation of peptidyl-tRNA [39,42]. In this study we found the following: first, RA-V specifically represses YAP reporter genes; second, RA-V inhibits the protein but not mRNA levels of endogenous YAP target genes. These results suggest that RA-V may inhibit Hippo pathway functions through suppressing the protein levels of its target genes. However, it is unclear why RA-V also exhibits specific inhibition of luciferase reporters driven by certain promoters. One possibility is that RA-V may affect the linked transcription–translation process, which is beyond the scope of this study.

Targeting protein synthesis has been explored as a direction for therapeutics for cancer. For example, temsirolimus and everolimus, inhibitors of the mTOR signaling pathway which governs protein synthesis, have been approved for the treatment of renal cell carcinoma [43]. However, inhibition of the mTOR pathway may have additional effects besides inhibition of protein synthesis. The choice of oncologic drugs directly targeting protein synthesis is still very limited. For example, Denileukin diftitox is an engineered protein combining Interleukin-2 and Diphtheria toxin, which inhibits translation elongation factor EF2. Denileukin diftitox could bind to Interleukin-2 receptor to be introduced into cells, and is approved for cutaneous T-cell lymphoma [44]. Omacetaxine mepesuccinate is another inhibitor of translation elongation and is approved for chronic myelogenous leukemia [45]. Thus, further identification of anticancer drugs with mechanisms of protein synthesis inhibition would be a promising direction. The potential of RA-V as an antitumor agent has been studied. For example, it exhibits cytotoxic activity on a panel of cancer cells such as liver cancer, breast cancer, and prostate cancer cell lines in the nanomolar range [46]. RA-V also represses the growth of subcutaneous tumor xenografts or transplanted leukemia cells in vivo [38]. However, these models may not be able to accurately reflect the antitumor activity of candidate compounds for many reasons, such as the lack of a proper microenvironment and metabolism of compounds. Thus, the evidence for RA-V as an anticancer agent falls short in that it has not been demonstrated to be effective for tumors grown orthotopically with an intact tumor microenvironment. Using two genetic models of liver cancer driven by deregulation of the Hippo pathway, we demonstrated that RA-V is not only able to repress tumorigenesis, but also able to ablate established tumors through mechanisms likely involving the normalization of the tumor immune microenvironment. These findings further support the potential use of RA-V as a drug against cancers, especially those involving deregulation of the Hippo pathway.

## 4. Materials and Methods

### 4.1. Cell Lines and Mice

HEK293T, HEK293A, HeLa and U2OS cell lines were from Dr. Kun-Liang Guan’s laboratory in the year 2012 and were cultured in Dulbecco’s Modified Eagle Medium (DMEM) (Life Technologies, Carlsbad, CA, USA) containing 10% fetal bovine serum (FBS) (Life Technologies) and 50 mg/mL penicillin/streptomycin at 37 °C in a humidified atmosphere of 5% CO_2_. Cell line authentication was not done in the lab.

All animal study protocols were approved by the Zhejiang University Animal Care and Use Committee. The animal protocol approval code is ZJU20170373. It is approved on 24 February 2017. *Mst1*^−/^^−^; *Mst2f/f* mice obtained from Dr. Yingzi Yang (Department of Developmental Biology, Harvard School of Dental Medicine, Boston, MA, USA) and Dr. Zengqiang Yuan (The Brain Science Center, Institute of Basic Medical Sciences, Beijing 100850, China; Center of Alzheimer’s Disease, Beijing Institute for Brain Disorders, Beijing, China.) were crossed with *Albumin*-Cre mice obtained from Dr. Yong Cang (Life Sciences Institute and Innovation Center for Cell Signalling Network, Zhejiang University, Hangzhou, Zhejiang, China.) for liver-specific *Mst1/2* double knockout. For hydrodynamic injection, four weeks old male ICR mice were purchased from the Shanghai SLAC Laboratory Animal Company. Plasmid DNA dissolved in sterile Ringer’s solution in a volume equal to 10% of the body weight was injected in 5–7 s via the tail vein. The amount of injected DNA was 50 μg of total transposon plasmids together with PB transposase plasmids. For bioluminescent imaging, mice were anaesthetized, and luciferin substrate was injected intraperitoneally. Signals were monitored by IVIS Lumina system (PerkinElmer, New York, NY, USA).

### 4.2. Chemicals and Antibodies

The 52,683 compounds library was from the National Center for Drug Screening in Shanghai, China. Cisplatin was purchased from Selleck (#S1166). RA-V was extracted and isolated from the root of *Rubia yunnanensis*. The details of extraction and isolation procedures for RA-V have been reported previously [46]. RA-V powder was stored at −20 °C as a stock. For in vitro cell studies, RA-V was dissolved in dimethyl sulfoxide (Sigma, Darmstadt, Germany) at 1.323 mM as a stock solution and stored at −20 °C. For in vivo mice studies, RA-V-loaded polymeric micelles were dissolved in PBS at 1 mg/mL before use and stored at 4 °C for a maximum of one week. RA-V-loaded micelles were prepared using thin-film dispersion method [38]. In brief, 10 mg RA-V and 200 mg mPEG2000-PDLLA2000 were completely dissolved in 100 mL mixed solvent (80 mL methylene chloride and 20 mL methanol) to obtain a clear solution in a round bottom flask. The solvent was removed by rotary evaporation under reduced pressure at 60 °C for about 3 h and a thin layer of uniform film on the wall of the flask was formed. Saline was added to the flask and the mixture was stirred for 10 min at 60 °C to prepare a RA-V containing polymeric micelle aqueous solution. Then the solution was passed through 0.22 μm filter membrane to remove the unincorporated RA-V aggregates. Information for antibodies is included in Table S1.

### 4.3. Screen for YAP Inhibitors

The primary screen was done using HEK293T stable cell clone expressing CTGF-luciferase reporter and Myc tagged-YAP. To establish the stable cell, HEK293T cells were first infected with pQCXIH-Myc-YAP and selected with hygromycin. Cells were then transfected with pGL4.21-CTGF-luciferase plasmid and selected with puromycin. Single clones were picked and luciferase activities were examined. Clone 4D10 with high reporter activity was used for the screen. During the screen, cells were seeded at 2500 cells/well in 384 well plates. Plates were incubated overnight and then compounds were added at 10 μg/mL by a robot. After 24 h, cells were lysed and luciferase activities were determined using the Luciferase Assay System (Promega, Madison, WI, USA) following the manufacturer’s instructions. Compounds in the primary screen were stored in two formats in 96 well plates: the regular plates have one compound/well; the XY plates have ten compounds/well and every compound is in two different wells. For compounds in XY plates, only when both wells containing the same compound meet the screen criteria, it was considered a positive hit. Positive hits from the primary screen were validated in a duplicate screen. Cell viability assay in the duplicate screen was carried out using CellTiter-Blue Assay Kit (Promega) following the manufacturer’s instructions.

The secondary screen was carried by transfecting HEK293T cells with Gal4-DBD-TEAD4, 9 × UAS-luciferase reporter, Flag-tagged YAP, and CMV-β-galactosidase. After 24 h, cells were seeded into 96 well plates at 25,000 cells/well, compounds were added at 10 μg/mL and 1 μg/mL, and after 24 h luciferase activities were determined using the Luciferase Assay System (Promega) following the manufacturer’s instructions. Luciferase activities were normalized to β-galactosidase activities.

### 4.4. Western Blotting

Western blotting was performed using standard protocol. Briefly, cells or mice tissue samples homogenized were lysed with 1% SDS lysis buffer (50 mM Tris-HCl added 1% sodium dodecyl sulfate, pH 6.8) and protein concentration was determined using the BCA Protein Assay Kit (Pierce, Waltham, MA USA). Protein samples were resolved on SDS-PAGE and then transferred to PVDF membranes. Membranes were blocked with 5% BSA (bovine serum albumin) and then incubated sequentially with primary and secondary antibodies, then washed. Protein expression was detected by ECL Detection Reagent (Pierce).

### 4.5. RNA Isolation and Real-Time PCR

Total RNA was isolated from cultured cells or mice tissue samples homogenized using Trizol Reagent (Life Technologies) following the manufacturer’s instructions. cDNA was synthesized by reverse transcription using random hexamers and was subjected to real-time PCR with gene-specific primers in the presence of SYBR Green Master Mix (Bio-Rad, Hercules, CA, USA). The relative abundance of mRNA was calculated by normalization to the level of hypoxanthine phosphoribosyltransferase 1 (HPRT) mRNA. Information for real-time PCR primers is included in Table S2.

### 4.6. Immunofluorescence Staining

For immunofluorescence staining, cells were cultured on coverslips coated with fibronectin to appropriate density. After incubated with vehicle or RA-V for 4.5 h, cells were fixed with 4% paraformaldehyde for 15 min and then permeabilized with 0.1% Triton X-100. After blocking in 3% BSA for 30 min, cells were incubated with first antibody diluted in 1% BSA for 1.5 h. After washing with PBS, slides were incubated with Alexa Fluor 488-or 594-conjugated secondary antibodies (1:1000) for 1.5 h. The slides were then washed and mounted with Prolong^™^ Gold antifade reagent (Life Technologies).

### 4.7. HE Staining, Sirius Red Staining, Immunohistochemistry and Multiplex Immunohistochemistry

Mouse livers were fixed in neutral buffered formalin for 24 h at room temperature and then paraffin embedded and processed according to standard protocols. Sections were deparaffinized through graded ethanol solutions.

For HE staining, after deparaffinization, sections were sequentially incubated in hematoxylin for 1 min, tap water for 5 min, 80% ethanol for 2 min, eosin for 10 sec, and then dehydrated quickly through graded ethanol solutions and hyalinized in xylene. Sections were then mounted in neutral balsam mounting media.

For Sirius red staining, after deparaffinization, sections were incubated in Sirius red for 1 h, washed and put in 100% ethanol for 5 min and hyalinized in xylene. Sections were then mounted in neutral balsam mounting media.

For immunohistochemistry, after deparaffinization, sections were heated to above 95 °C in appropriate antigen retrieval solution for 30 min. After cooling down, sections were stained with specific antibodies using the avidin–biotin complex system (Vector Laboratories). 3,3′-diaminobenzidine (DAB) was used as the substrate. Cell nuclei were counterstained with hematoxylin. Sections were then mounted in neutral balsam mounting media.

Multiplex immunohistochemistry was carried out using Opal Multiplex IHC Detection Kit (PerkinElmer) following the manufacturer’s instructions.

### 4.8. Flow Cytometry

To isolate mouse liver tumor lymphocytes and hepatocytes, tumors were cut into small pieces and digested in 10 mL RPMI 1640 medium (Life Technologies) containing 50 mg/mL penicillin/streptomycin, 0.1% collagenase type I, 0.15% collagenase type II, 0.2% collagenase type IV and 1 µL DNase for 1 hour. Cell suspensions were passed through a 100 µm cell strainer (Corning, Corning, NY, USA) and centrifuged at 50× *g* for 5 min at 4 °C. The suspension is for immune cell isolation, while the pellet is for hepatocyte isolation.

To isolate hepatocytes, the pellet was suspended in DMEM medium and centrifuged at 50× *g* for 12 min at 4 °C. Then the pellet was suspended again in 5 mL sterile PBS and mixed with 5 mL 100% Percoll, centrifuged at 50× *g* for 10 min. Pellet was washed, suspended using DMEM medium containing 50 mg/mL penicillin/streptomycin and 10% FBS, then counted, diluted and seeded at 250,000 cells/well into 6 well plates.

To isolate immune cells, the cell suspension was passed through a 70 µm cell strainer (Corning) and centrifuged at 300× *g* for 5 min at 4 °C. The cell pellet was resuspended in 2 mL PBS to generate a single-cell suspension. A centrifuge tube was layered from the bottom with 50% Percoll/PBS, 25% Percoll/PBS, and the single-cell suspension, then centrifuged at 1300× *g* for 20 min. The cell layer between 25% and 50% Percoll/PBS was collected, washed once with the RPMI 1640 medium. Pellet was washed, suspended using RPMI 1640 medium containing 50 mg/mL penicillin/streptomycin and 10% FBS, then counted, diluted and seeded at 500,000 cells/well into 6 well plates.

Then the hepatocytes and immune cells were incubated with DMSO, RA-V or cisplatin for 12 h. Cells were then collected by centrifugation at 500× *g* for 5 min. Cells were processed for Annexin V-PE Apoptosis Detection Kit (BD, Franklin Lakes, NJ, USA) staining and flow cytometry following the manufacturer’s instructions. Data was analyzed by FlowJo software (Ashland, OR, USA).

### 4.9. Statistical Analysis

Quantification of staining was done by ImageJ software (https://imagej.nih.gov/ij/). *P* values were determined by Student’s *t* test.

## 5. Conclusions

In conclusion, cyclopeptide RA-V blocks liver enlargement and tumorigenesis induced by inactivation of the Hippo pathway. Furthermore, RA-V caused regression of orthotopic mouse liver tumors induced by YAP. Mechanistically, RA-V inhibits proliferation and promotes apoptosis of both tumor cells and immune cells in the tumor microenvironment. These findings warrant further investigation of RA-V as a drug candidate for cancers involving YAP/TAZ activation.

## Supplementary Materials

The following are available online at http://www.mdpi.com/2072-6694/10/11/449/s1, Figure S1: Establishment of YAP reporter cell line, Figure S2: RA-V represses the protein levels of YAP target genes, Figure S3: *Mst1/2* double knockout induces liver enlargement, Figure S4: Short-term RA-V treatment did not significantly affect liver/body weight ratio, Table S1: List of Antibodies for WB and IHC Analysis, Table S2: List of primers for qPCR analysis.
