# Supplementary material for: Cyclopeptide RA-V Inhibits Organ Enlargement and Tumorigenesis Induced by YAP Activation
Source: Cancers (Basel). 2018 Nov 16;10(11):449. doi: 10.3390/cancers10110449 (PMC6265802; doi:10.3390/cancers10110449)
Supplement: Supplementary file 1 [file cancers-10-00449-s001.pdf]

Supplementary Materials: Cyclopeptide RA-V Inhibits Organ Enlargement and Tumorigenesis Induced by YAP Activation

Xinyan Ji, Lihua Song, Li Sheng, Anhui Gao, Yang Zhao, Shixun Han, Yuchao Zhang, Chu Zhu, Simeng Zhao, Zhe Wang, Bohan Xu, Li Li, Jia Li, Ninghua Tan and Bin Zhao

Supplementary Figures

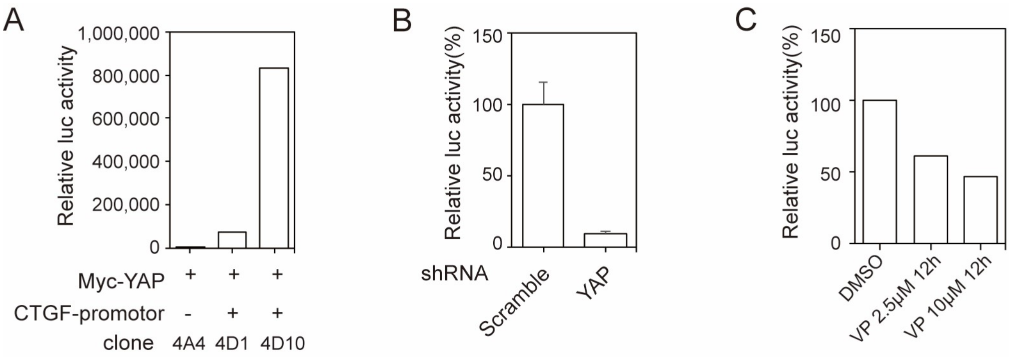

Figure S1. Establishment of YAP reporter cell clone. (**A**) HEK293T clones stably expressing YAP and a luciferase reporter driven by the promoter of CTGF. (**B**) Knockdown of YAP inhibited reporter activity in cell clone 4D10 from (A). 4D10 cells were infected with scramble or shRNA against YAP. Luciferase activity was then determined. (**C**) Verteporfin (VP) inhibited reporter activity in cell clone 4D10 from (A). 4D10 cells were treated with VP as indicated. Luciferase activity was then determined.

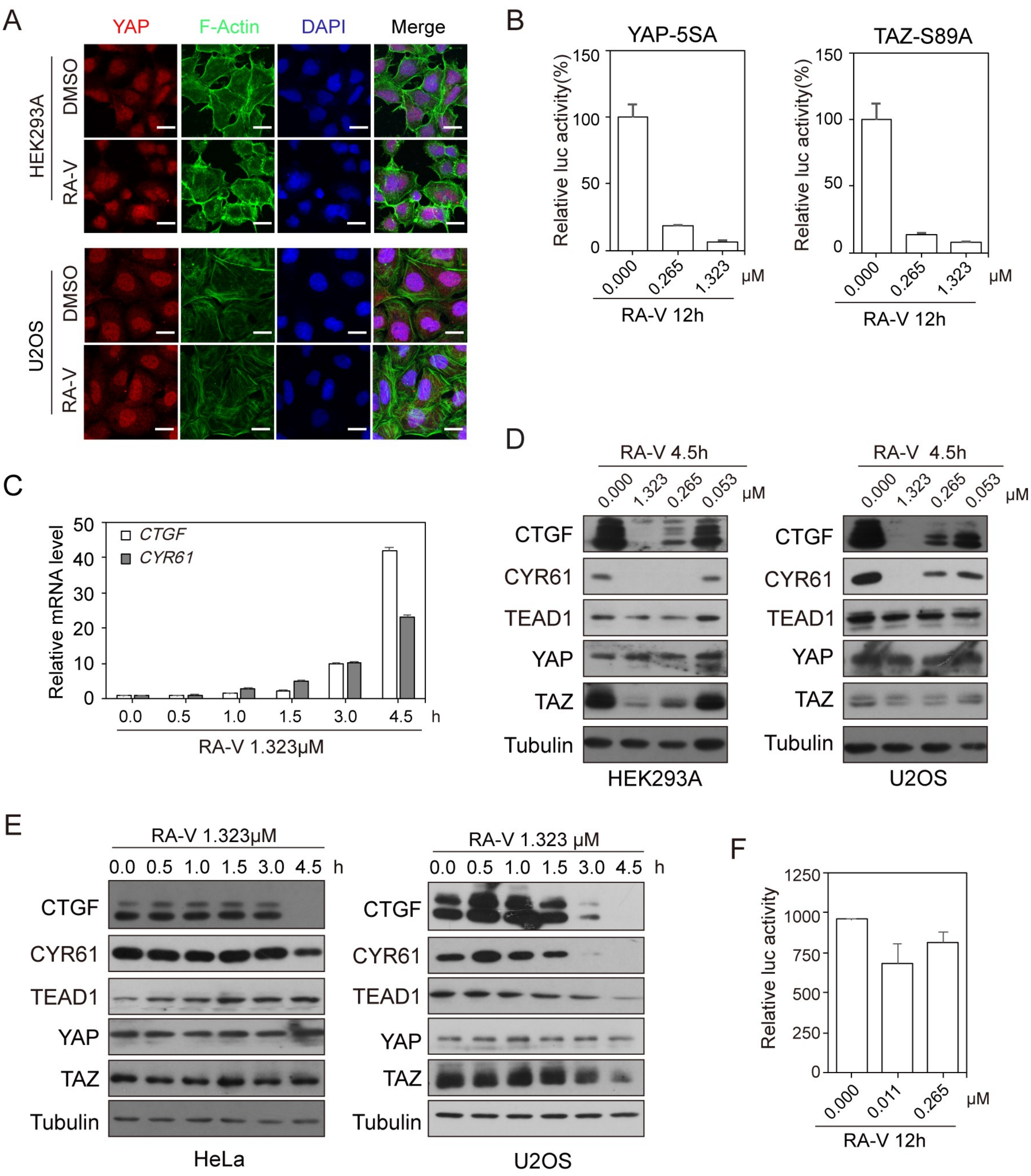

Figure S2. RA-V represses the protein levels of YAP target genes. (**A**) RA-V does not affect YAP subcellular location. HEK293A and U2OS cells were trypsinized and attached again in the presence of DMSO or RA-V at 0.265 μM for 4.5 h. Cells were then subjected to immunofluorescence staining. Scale bars, 20 μm (**B**) RA-V inhibits luciferase activity induced by YAP-5SA or TAZ-S89A mutants. HEK293T cells were transfected with YAP-5SA or TAZ-S89A, Gal4-TEAD4, 9 × UAS-luciferase, β-galactosidase and treated as the indicated for luciferase activity. (**C**) RA-V induces mRNA expression of *CTGF* and *CYR61* in HeLa cells. Experiments were similar to these in Figure 2E. (**D** and **E**) The protein levels of CTGF and CYR61 were repressed by RA-V in a dose-(D) and time-(E) dependent manner. HEK293A, U2OS and HeLa cells were trypsinized and attached again for 4.5 h in the presence of DMSO or RA-V. Cells were then lysed and examined by western blotting. (**F**) RA-V does not inhibit a Wnt reporter gene. HEK293T cells were transfected with TOP-flash reporter gene, active β-catenin-ΔN mutant and β-galactosidase. Cells were treated as the indicated for luciferase activity assay.

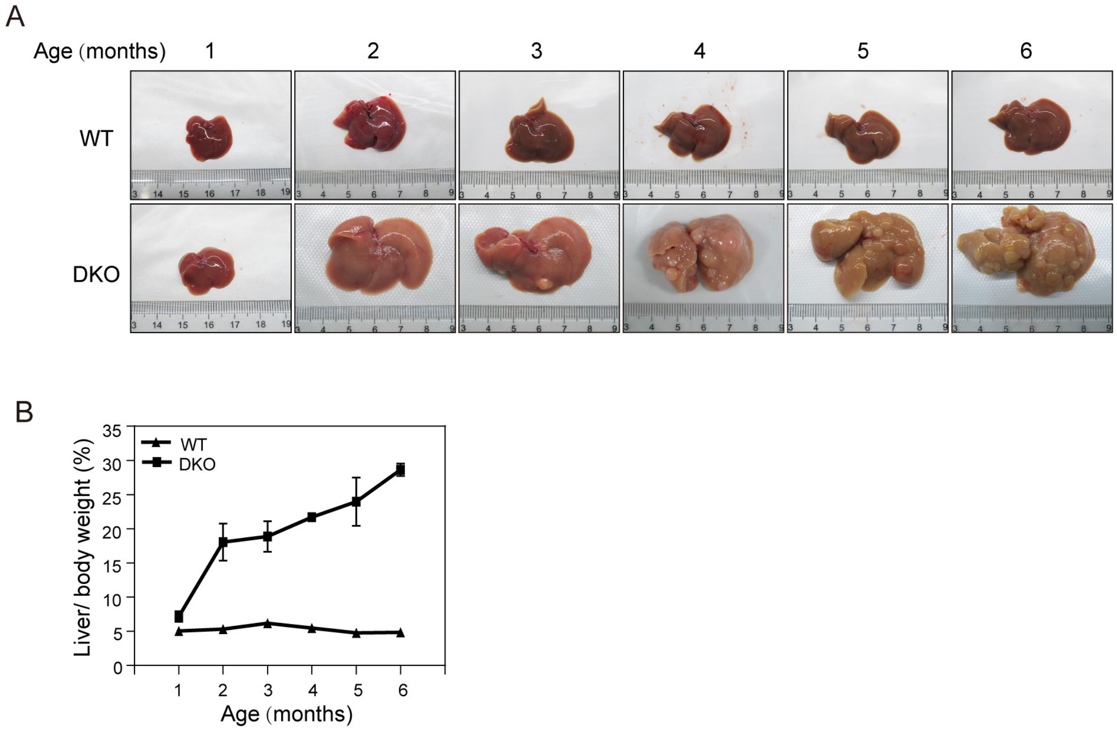

Figure S3. *Mst1/2* double knockout induces liver enlargement. (**A**) Representative pictures of livers from mice at the indicated age. (**B**) Quantification of liver/body weight ratios ($n = 4$). Values represent mean ± SD.

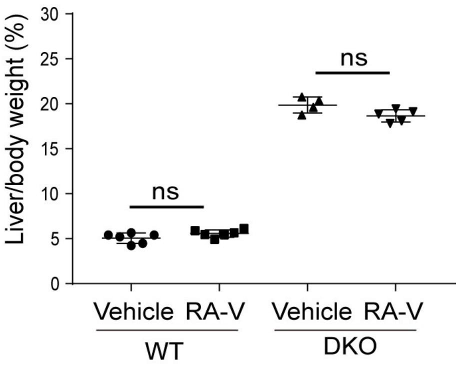

Figure S4. Short-term RA-V treatment did not significantly affect liver/body weight ratio. Liver/body weight ratio of wild-type (WT) and *Mst1/2* DKO mice treated with vehicle or RA-V (5 mg/kg) as the schedule shown in Figure 4A. Values represent mean ± SD with all data points plotted. *p* values were determined by Student's *t* test. ns, not significant.

Supplementary Tables

Table S1. List of Antibodies for WB and IHC Analysis.

No.	Antibody	Manufacturer	Catalog Number
1	α-tubulin	Sigma	#T5326
2	YAP	Santa Cruz	#sc-15407
3	Phospho-YAP S127	Cell Signaling Technology	#4911
4	TEAD	BD	#610923
5	TAZ (V386)	Cell Signaling Technology	#4883
6	CTGF	Santa Cruz	#sc-14939
7	CYR61	Santa Cruz	#sc-13100
8	LATS2	Bethyl	#A300-479A
9	Hsp90	BD	#610418
10	Flag-tag	Sigma	#F7425
11	Myc-tag	Cell Signaling Technology	#2276
12	HNF4a	PPMX	#PP-H1415-00
13	phospho-Histone H3	Cell Signaling Technology	#9701
14	Angiomotin	Bethyl	#A303-305A
15	Keratin 17/19	Cell Signaling Technology	#12434
16	Cleaved Caspase3	Cell Signaling Technology	#9664
17	Ki67	Dako	#M7249
18	Ki67	Abcam	#ab16667
19	CD45	eBioscience	#11-0451
20	Active YAP	Abcam	#R19812
21	Phospho-YAP (Ser127)	Cell Signaling Technology	#S13008

Table S2. List of primers for qPCR analysis.

Primer	Primer Sequence
hHPRT-RT-F	ACTGTAATGATCAGTCAACGGG
hHPRT-RT-R	GGCCTGTATCCAACACTTCG
hCTGF-RT-F	CCAATGACAACGCCTCCTG
hCTGF-RT-R	TGGTGCAGCCAGAAAGCTC
hCYR61-RT-F	AGCCTCGCATCCTATACAACC
hCYR61-RT-R	TTCTTTCACAAGGCGGCACTC
hAMOTL2-RT-F	GGACACCCTCTCTGGACTCT
hAMOTL2-RT-R	GAAGACAACTGCCGGAATG
hANKRD1-RT-F	AGCGAGAAACAACGAGAGG
hANKRD1-RT-R	CATCCACAGGTTCCGTAATG
hDIAPH3-RT-F	TCTGCGGTATGCATTGTAGGG
hDIAPH3-RT-R	TGAACTGAATTGTGCCGGAG
hBIRC5-RT-F	CATTCGTCCGGTTGCGC
hBIRC5-RT-R	GGCGCACTTTCTCCGCAG
hFOXM1-RT-F	CACAGCATCATCACAGCAC
hFOXM1-RT-R	GGTCTCCAGGGTCACTTCT
hITGB2-RT-F	CCCGACACCCTGAAAGTCAC
hITGB2-RT-R	GCTCCTGGATGCACTCTCTGTG

© 2018 by the authors. Licensee MDPI, Basel, Switzerland. This article is an open access article distributed under the terms and conditions of the Creative Commons Attribution (CC BY) license (http://creativecommons.org/licenses/by/4.0/).
